# Atorvastatin ameliorates cerebral vasospasm and early brain injury after subarachnoid hemorrhage and inhibits caspase-dependent apoptosis pathway

**DOI:** 10.1186/1471-2202-10-7

**Published:** 2009-01-21

**Authors:** Gao Cheng, Liu Wei, Sun Zhi-dan, Zhao Shi-guang, Liu Xiang-zhen

**Affiliations:** 1 Department of Neurosurgery, The First Affiliated Hospital of Harbin Medical University, No.23 Youzheng Street, Nangang District, Harbin, Heilongjiang, PR China; 2 Department of Cardiology, The First Affiliated Hospital of Harbin Medical University, No.23 Youzheng Street, Nangang District, Harbin, Heilongjiang, PR China

## Abstract

**Backgroud:**

Cerebral vasospasm (CVS) and early brain injury remain major causes of morbidity and mortality after aneurysmal subarachnoid hemorrhage (SAH). Hydroxymethylglutaryl coenzyme A reductase inhibitors, also known as statins, has the neuroprotective effects and ameliorating CVS after SAH. This study was designed to explore apoptosis inhibiting effects of atorvastatin and its potential apoptotic signal pathway after SAH.

**Results:**

Preserving blood-brain-barrier permeability, decreasing brain edema, increasing neurological scores and ameliorating cerebral vasospasm were obtained after prophylactic use of atorvastatin. TUNEL-positive cells were reduced markedly both in basilar artery and in brain cortex by atorvastatin. Apoptosis-related proteins P53, AIF and Cytochrome C were up-regulated after SAH, while they were not affected by atorvastatin. In addition, up-regulation of caspase-3 and caspase-8 after SAH was decreased by atorvastatin treatment both in mRNA and in protein levels.

**Conclusion:**

The neuroprotective effects of atorvastatin after SAH may be related to its inhibition of caspase-dependent proapoptotic pathway based on the present results.

## Background

Aneurysmal subarachnoid hemorrhage (SAH) affects 10 per 100 000 population in the Western world. For survivors of the initial hemorrhage, cerebral vasospasm and early brain injury are major causes of subsequent morbidity and mortality [1]. Apoptosis has even been demonstrated taking part into aneurismal formation and post SAH vasospasm and early brain injury [2,3]. Following the global ischemia seen with SAH, apoptosis has been shown to occur in the hippocampus, blood-brain barrier (BBB), and vasculature with varying degrees of necrosis [4]. Several apoptotic pathways that are believed to be involved in SAH, including the death receptor pathway, caspase-dependent and-independent pathways, and the mitochondrial pathway [5].

A growing body of clinical and experimental literature demonstrates that statins have neuroprotective effects on stroke but the mechanism(s) by which these drugs improve stroke outcome is still unclear [1]. Increasing evidences, however, link these effects to their cholesterol-independent properties since statins reduce vascular inflammatory responses, ameliorate endothelial function, and modulate cytokine responses and NOS activity [6]. The putative neuroprotective actions of statins may lead to functional restoration after SAH. However, the effects of statins in the SAH paradigm are not well known till now. In the present study, we investigate whether atorvastatin, when administered prophylactically, can reduce brain edema formation, cerebral vasospasm, cell death, and subsequently promote neurological recovery in a rat model of SAH. Three recognized apoptotic pathways were examined, the caspase-dependent and caspase-independent pathways and the mitochondrial pathway. Cytochrome C was chosen to represent the mitochondrial pathway, apoptosis-inducing factor (AIF) was chosen to represent the caspase-independent pathway, and caspases 3 and 8 were chosen to represent the caspase-dependent pathway. P53 was also been determined as it has been demonstrated playing an orchestrating role in apoptotic cell death after experimental SAH [7]. By examining these apoptosis-related proteins, we hoped to provide an overview of atorvastatin on apoptotic pathways after SAH.

## Results

### Physiological data and mortality

No obvious difference in physiological data was found among groups at baseline. The blood pressure rose abruptly just after puncture of ICA and decreased to normal level at about 5 mins (data not shown), which was consistent with previous report [8] and our previous results [9]. The mortality at 24 hour was 50.0% (8 of 16) in SAH + vehicle group, 25.0% (4 of 16) in atorvastatin treated group, 43.8% (7 of 16) in SAH group and none in SC group (0 of 8). The reduction in mortality with atorvastatin treatment was significant lower than that in vehicle treated group (*P *< 0.05). No significant difference was found in extent of SAH between atorvastatin and DMSO group at autopsy (*P *> 0.05).

### Cerebral vasospasm

The mean cross-sectional area of BA was 8281 ± 748 μm^2 ^in SAH + atorvastatin rats, versus 5405 ± 493 μm^2 ^in SAH+DMSO group, 5874 ± 587 μm^2 ^in SAH group and 9012 ± 843 μm^2 ^in SC group (atorvastatin group versus DMSO group, *P *< 0.05; ANOVA). The mean wall thickness of BA was 16.50 ± 5.23 μm in SAH+ atorvastatin group, 28.50 ± 7.24 μm in SAH+DMSO group, 27.13 ± 6.33 μm in SAH group and 14.24 ± 3.21 μm in SC group (atorvastatin group versus DMSO group, *P *< 0.05; ANOVA).

### Neurological scores

The neurological scores of rats in atorvastatin group were significantly lower (*P *< 0.05; ANOVA) than that in sham-operated group at 6 hour after SAH (14.1 ± 2.9 versus 18.0 ± 0.4). And atorvastatin did not improve neurological functions at 6 hour. However, neurological scores were improved at 24 hour after SAH in the atorvastatin treated rats, which were closed to the sham operated rats(17.3 ± 3.7 versus 18.0 ± 0.5, *P *> 0.05).

### BBB permeability

In SAH animals, marked extravasation of Evan's blue dye into all brain regions was observed at 24 hour, especially in both hemispheres. High values of Evan's blue dye were obtained in brain stem and cerebellum, although no statistical significance was observed between the two regions. Treatment with atorvastatin significantly decreased the amount of Evan's blue extravasation both in hemispheres and in brain stem (1.58 ± 0.23 μg/g in atorvastatin group versus 1.23 ± 0.14 μg/g brain tissue in DMSO group, *P *< 0.05, ANOVA).

### Brain water content

Significant increase in brain water content was detected in SAH rats at 24 hour compared with that in sham operated control rats (78.45 ± 3.23% versus 73.02 ± 2.45%, *P *< 0.05). Water content was decreased significantly by atorvastatin as compared with that in DMSO group. (74.12 ± 3.18% versus 78.23 ± 3.02%, *P *< 0.05).

### TUNEL staining and cell death assay

**Figure 1:**
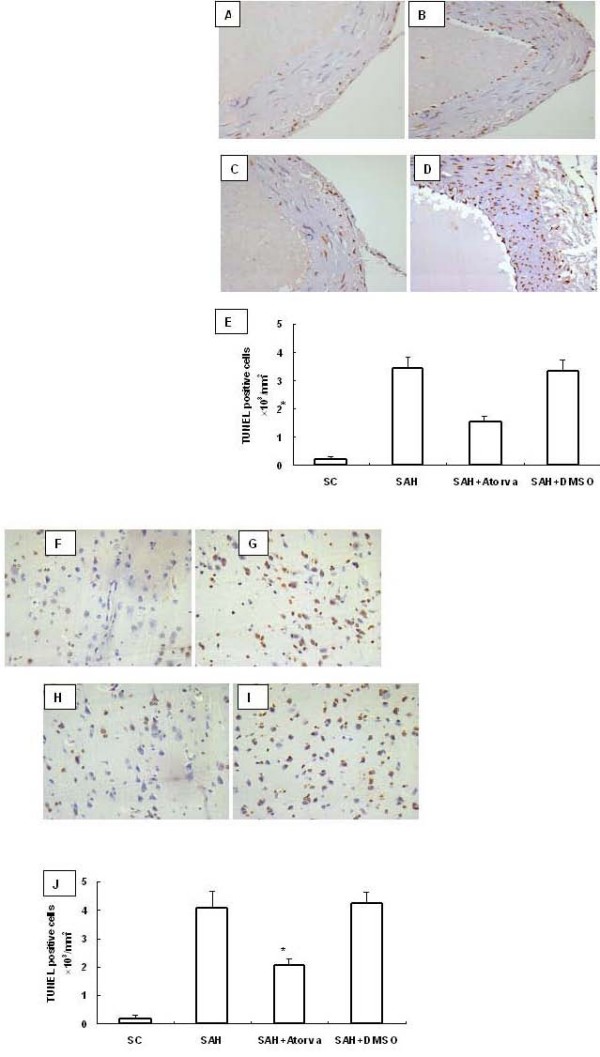
**TUNEL staining of basilar artery and basal cortex after SAH**. Few apoptosis cells were observed in sham operated control rats (A and F). Increased TUNEL positive staining was observed in SAH+DMSO (B and G) and SAH rats (D and I), which was reduced markedly by atorvastatin. (C and H). Scale bar represents 200 μm. Quantitive analysis showed an obvious reduction in number of apoptosis cells (number/mm^2^) by atorvastatin compared with that in the DMSO group. (E and J, *P *< 0.01 vs DMSO group).

Few TUNEL positive cells were detected in sham operated animals. At 24 hour after SAH, TUNEL positive cells appeared both in basilar artery and in basal cortex in SAH rats. Treatment with atorvastatin produced remarkable reduction of TUNEL positive staining both in basilar artery and in brain cortex (Figure 1A–J).

**Figure 2:**
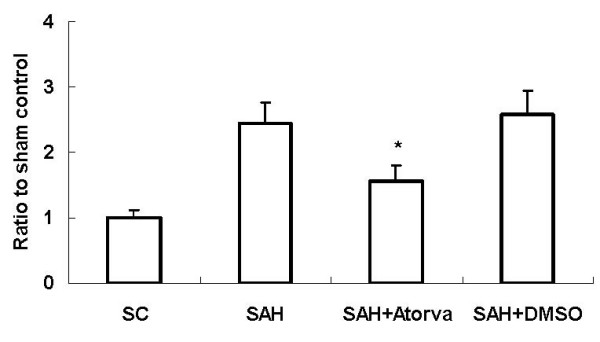
**Cell death assay of DNA-fragmentation after SAH**. The results showed that apoptotic-related DNA-fragmentation was increased significantly in SAH rats compared with that in SC rats at 24 hour in basal cortex. Atorvastatin decreased the DNA-fragmentation markedly (*, *P *< 0.05 vs DMSO group).

To investigate apoptotic cell death in acute brain injury after SAH, DNA fragmentation was analyzed with a commercial enzyme immunoassay (cell death assay). In the SAH group, the cell death assay revealed that DNA fragmentation was significantly increased at 24 hour compared with the sham operated rats. DNA fragmentation was significantly decreased in atorvastatin-treated rats compared with that in the vehicle-treated rats at the same time point (Figure 2).

### Apoptosis-related proteins expression

**Figure 3:**
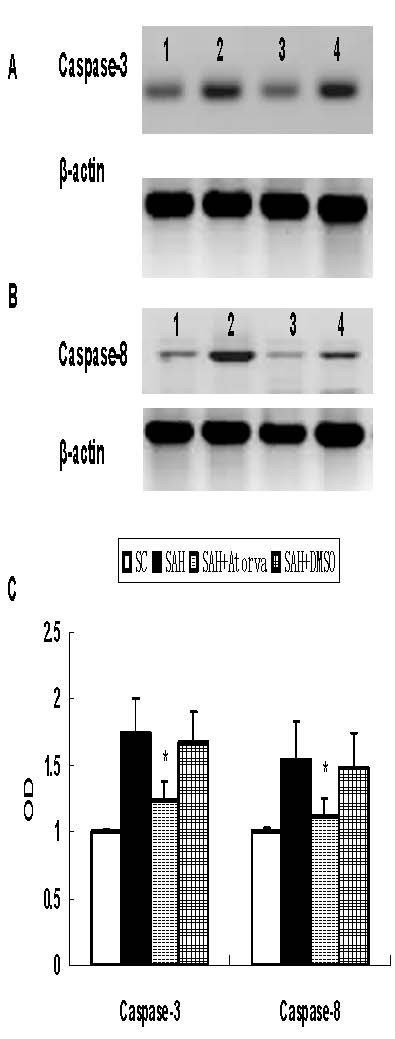
**Western blot for expression of caspase-3 and caspase-8**. Obvious increase of caspase-3 (P17) and caspase-8 (P18) was obtained in basal cortex, which was attenuated by atorvastatin. A, Represent expression of caspase-3. B, Represent expression of caspase-8. C, Each column represents 5 independent experimental results. (1, Sham operated control; 2, SAH; 3, SAH + Atorvastatin; 4, SAH+DMSO *, *P *< 0.05 vs DMSO group).

**Figure 4:**
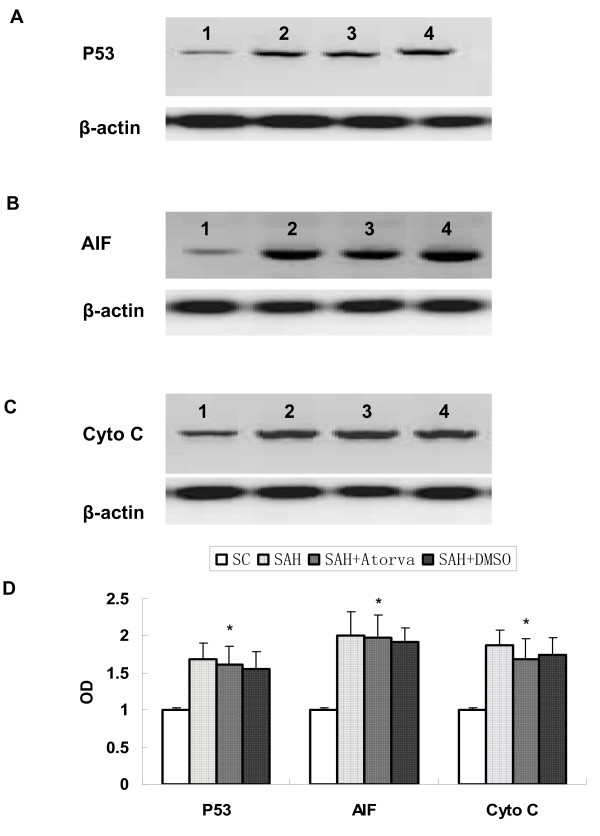
**Represent Western blot for expression of P53 (A), AIF (B), and Cytochrome C (C) in brain cortex**. Obvious increase of these apoptosis-related proteins was observed after SAH, while they were not affected by atorvastatin-treatment. Each column represents 5 independent experimental results (D). (1, Sham operated control; 2, SAH; 3, SAH+Atorvastatin; 4, SAH + DMSO. *, *P *= ns vs DMSO group).

**Figure 5:**
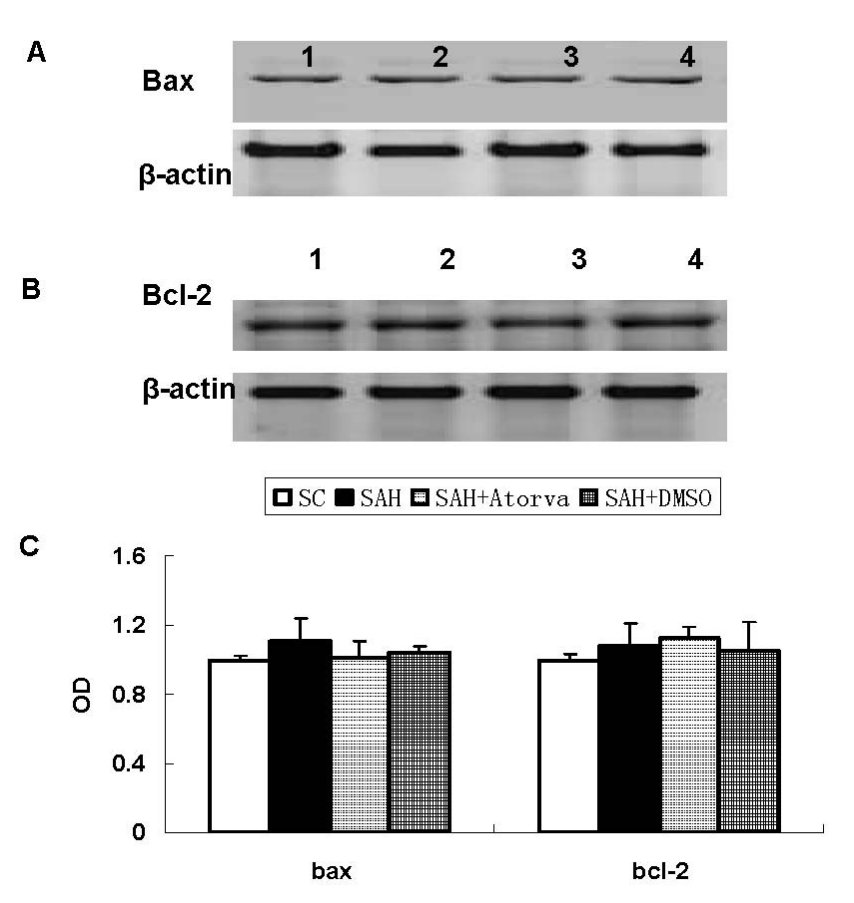
**Represent Western blot for expression of bax (A) and bcl-2 (B) in brain cortex**. Bax and bcl-2 proteins expression was not changed after SAH, and they were not affected by atorvastatin treatment. C, Each column represents 5 independent experimental results. (1, Sham operated control; 2, SAH; 3, SAH+Atorvastatin; 4, SAH+DMSO).

Apoptosis-related proteins were examined in hippocampus and basal cortex. Elevated level of cleaved caspase-3, caspase-8, AIF, P53 and Cytochrome C were observed in SAH group (*P *< 0.05 versus sham operated control group). While no significant difference was found of bax and bcl-2 proteins levels between SAH and sham operated control rats. Atorvastatin reduced the expression of cleaved caspase-3 and caspase-8 (*P *< 0.05 versus vehicle treated rats), but had no obvious influence on expression of AIF, P53 and Cytochrome C. Atorvastatin had no statistical effects on both bax and bcl-2 at 24 hour after SAH (Figure 3, 4, 5).

### mRNA expression of caspase-3 and caspase-8

**Figure 6:**
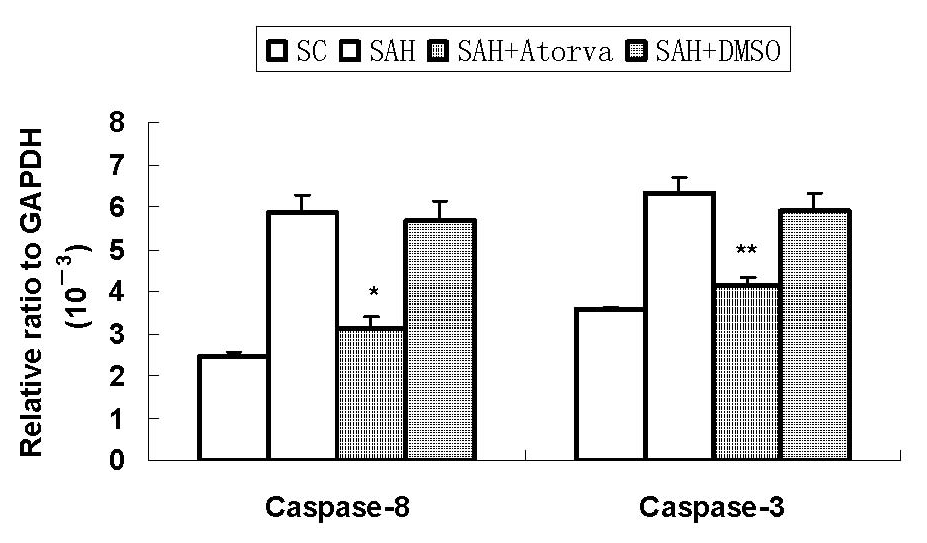
**mRNA expression of caspase-3 and caspase-8**. Consistent with the Western blotting results, mRNA caspase-3 and caspase-8 was significantly upregulated after SAH. It was significantly reduced by atorvastatin. (*, *P *< 0.05 and **, *P *< 0.01 vs DMSO group).

Consistent with the Western blotting results, mRNA expression of caspase-3 and caspase-8 was increased after SAH, and it was decreased markedly by atorvastatin treatment at 12 h after SAH (Figure 6).

## Discussion

In this study, we demonstrated that the pleiotropic 3-hydroxyl-3-methyl-glutaryl-coenzyme A (HMG-CoA) reductase inhibitor, atorvastatin, when administered prophylacticly, ameliorates early brain injury and cerebral vasospasm after experimental SAH. Inhibition of the caspase-dependent apoptotic pathways, while not other apoptosis-related proteins, was observed after treated with atorvastatin. The neuroprotective effects may be involved in its potential anti-apoptosis mechanisms. Administration of caspase inhibitors has been found to reduce caspase-3 activation after SAH [5,10]. However, in the present research, atorvastatin, which is not a direct inhibitor of caspase, significantly reduced caspase-3 activation. It has been documented that P53 plays an orchestrating role in apoptotic cell death after experimental SAH [7]. Expression of P53 and caspase-3, 8 was decreased by P53 inhibitor, Pifithrin-α, which showed that P53 acting as an upstream of caspase activation. However, expression of P53 and other apoptosis-related proteins were not influenced by atorvastatin based on our results. It can be speculate that, at least in the apoptosis pathway, atorvastatin has the direct effect on caspase-3. Pleiotropic effects of statins has been found after SAH, such as increased phosphorylation of Akt and endothelial nitric oxide synthase [11]. However, whether anti-apoptotic effect of atorvastatin via these pathways was not determined in the present research.

Despite intense research efforts in aneurismal SAH, we currently possess a very limited understanding of the underlying mechanisms that result in brain injury [12]. However, a number of studies have recently indicated that apoptosis may be a major player in the pathogenesis of secondary brain injury after SAH [13]. As a result, the apoptotic cascades present a number of potential therapeutic opportunities that may ameliorate secondary brain injury. Experimental data suggest that these cascades occur very early after initial insult and may be related directly to physiologic sequela commonly associated with SAH [13]. In accordance with the previous reports, our data imply that numbers of TUNEL positive cells are significantly increased in SAH rats, while atorvastatin administered ahead markedly inhibited apoptosis after SAH.

Apoptosis requires activation of caspases, a cascade of cysteine proteases activated by proteolytic cleavage, which target key homeostatic and structural proteins, such as actin and fodrin as well as nuclear proteins like poly (ADP-ribose) polymerase (PARP), leading to DNA fragmentation and cell death. Caspase-3 activation is considered one of the final steps responsible for apoptosis [10]. Caspase-3 can proteolytically cleave several crucial cellular proteins inducing the characteristic changes associated with apoptosis including PARP-1, considered a hallmark biochemical characteristic of apoptosis. PARP-1 cleavage facilitates DNA alteration and nuclear disassembly and ensures the completion of the energy-dependent apoptotic process. In this study, atorvastatin significantly reduces the increased caspase-3 protein expression observed 24 hour after the insult. Although several studies have shown that statins may cause apoptosis in different cell lines, including neuronal cells [14,15], our data show that administration of atorvastatin to rats reduces the caspase-dependent apoptotic signal induced by SAH. In agreement with our results, it has been recently reported that another statin, pravastatin, reduces apoptosis in the hippocampus of adult rats after transient forebrain ischemia [16]. The reason for the different effect observed after "in vitro" or "in vivo" exposure to statins is unclear. One possibility is that statins do not exert a proapoptotic effect on brain cells "in vivo". This hypothesis is supported by the observation that caspase-3 activation and PARP cleavage were reduced in simvastatin-treated control animals or in the contralateral side of simvastatin-treated ischemic animals [17]. Another possibility is that the pro-apoptotic effect on inflammatory cells and the inhibition of leucocyte function induced by statins perhaps overcomes the potential pro-apoptotic effect they might have on neuronal and/or glial cells and the ultimate result is neuroprotection. Hydroxymethylglutaryl coenzyme A reductase inhibitors have an attractive profile for cerebrovascular research. Statins have been demonstrated to reduce stroke events independent of their lipid-lowering effect as well as to reduce mortality significantly after myocardial infarction [18]. We report here that prophylactic treatment with atorvastatin reduces caspase-3 and caspase-8 expression suggesting that its neuroprotective effect may result from a reduced number of neurons undergoing apoptotic cell death. To date, the apoptotic pathways believed to play a major role in SAH-induced apoptosis are the death receptor and TNF-a, p53, and the caspase dependent cascades [19]. However, to the best of our knowledge, multiple level inhibitors have not been used. An example of this might be to selectively block the limiting steps of the caspase independent and -dependent cascades and the bcl-2 family proteins. But in our present study, protein levels of bcl-2 and bax expression did not differ statistically among the three groups, suggesting that bcl-2 pathway may not participate into the apoptosis involved in experimental SAH. As to p53, AIF, and Cytochrome C, our data indicated an upregulation of these apoptosis-related proteins after SAH. However, it was not affected by atorvastatin, the underlying mechanisms need further investigation. P53 was thinked as a key molecular mediated apoptosis after SAH. Inhibition of P53 with PFTα down-regulated expression of other apoptosis-related proteins, including caspase-3 and caspase-8 [7]. In this research, P53 was not inhibited obviously by atorvastatin. This suggested that a unique mechanism of apoptosis inhibition underlying in atorvastatin, which made it more safety for treating SAH than other multi-levels apoptosis inhibitors.

Effects of statins on cerebral vasospasm and neurological outcome after SAH were still controversial [20,21]. Ameliorating cerebral vasospasm, improving cerebral autoregulation, and reducing vasospasm-related DID of statins were observed in some researches [20]. But based on the more recent report, statins using was not associated with less vasospasm and improved outcome after SAH [21]. Difference in enrolled patients, research methods, and outcome measurement may explain the contradictions. The contradictions highlighted the importance of basic study and clinical trials for statins after SAH. As apoptosis had been observed in hippocampus, blood-brain barrier (BBB), and vasculature in SAH patients [4], apoptosis inhibition proved by the present research may be partly elucidate the effects of long-term neurological improvement of statins. Based on the current research, statins may be beneficial for SAH treatment due to its effects of ameliorating cerebral vasospasm and improving neurological outcome.

## Conclusion

In summary, it appears that cerebral vasospasm and early brain injury after SAH are combination of physiologic insults to the brain, resulting in global ischemia, BBB breakdown, brain edema, and cellular death signaling. Atorvastatin ameliorated the vasospasm and early brain injury after experimental SAH. The underlying mechanisms may be related to its inhibition of caspase-dependent proapoptosis pathway.

## Methods

The protocol for these experiments was approved by the special committee on Animal Welfare of Harbin Medical University and all animals were treated humanely in accordance with the guidelines for the Care and Use of laboratory Animals published by the U.S. National Institutions of Health (NIH Publication No. 85-23, revised 1996).

### SAH rat model and grouping

Perforating SAH model was induced as reported previously with slight modifications [22]. Male Sprague-Dawley rats (300 to 350 g, bought from Harbin Veterinarians Research Institution) were anesthetized with 2.0% isoflurane in 70% nitrous oxide and 30% oxygen using a face mask. SAH was confirmed by autopsy in each rat. Animals that died during surgery were excluded from the experiment. Animals that died after surgery were replaced until the final group size reached expected number in each group.

Atorvastatin or same volume of vehicle (dimethyl sulfoxide, DMSO) was administered orally by gastric gavage for 15 days before operation. Atorvastatin was dissolved in 1% DMSO and further diluted in physiological buffer solution (PBS, final 0.01% DMSO). 20 mg·Kg^-1^·d^-1 ^of atorvastatin was selected because of stroke prevention effect was obtained previously in this dose [23]. In sham operated control (SC) and SAH group (SAH), no treatment was applied.

### Mortality and extent of SAH

Mortality was calculated at 24 hour after SAH. The number of rats used in each group was SAH+atorvastatin (n = 16), SAH+DMSO (n = 16), SAH (n = 16) and SC (n = 8). The animals were sacrificed and autopsied after operation and extent of SAH was evaluated according to the method reported by Sugawara et, al [24].

### Neurological deficits

Neurological deficits were evaluated at 6 and 24 hour after SAH using scoring system reported by Garcia et, al. with slight modifications [25]. The minimum neurological score is 3 and the maximum is 18. The number of rats used in each group was SAH+ atorvastatin (n = 8), SAH+DMSO (n = 8), SAH (n = 6), and SC (n = 6) before sacrificed. Two independent investigators (LW and SD) blinded to the grouping took the measurements.

### Brain water content

The entire brain was removed at 24 hour and weighed immediately (wet weight) and again after drying in an oven at 105°C for 24 hours (dry weight). The percentage of brain water content was calculated as [(wet weight - dry weight)/wet weight] × 100%. The number of rats used in each group was SAH+ torvastatin (n = 5), SAH+DMSO (n = 5), SAH (n = 5), and SC (n = 5).

### BBB permeability

According to the protocol of Uyama et al [26]. and the extraction method by Rossner and Temple [27], BBB permeability was assessed at 24 hour after SAH. Evan's blue dye (2%; 5 mL/kg) was injected over 2 minutes into the right femoral vein and allowed to circulate for 60 minutes. The amount of extravasated Evan's blue dye into the brain was determined by spectrofluorophotometry. Measurements were conducted at excitation wavelength 620 nm, emission wave length 680 nm, and bandwidth 10 nm. The number of animals used in each group was SAH+ atorvastatin (n = 5), SAH+DMSO (n = 5), SAH (n = 5) and SC (n = 5).

### Histological examination and cross-sectional area of BA measurement

At 24 hour after SAH, rats were anesthetized and intracardially perfused with PBS followed by 4% paraformaldehyde as described previously [4]. Brains were removed and postfixed in 4% paraformaldehyde for 3 days. Sections were embedded in paraffin and sliced into 6 μm and then stained with hematoxylin and eosin (H-E). The cross-sectional area of basilar artery was calculated as described by others and our laboratory previously [9,22]. An independent investigator blinded to the grouping took the measurements. The number of animals used in each group was SAH+ atorvastatin (n = 5), SAH+DMSO (n = 5), SAH (n = 5), and SC (n = 5).

### TUNEL staining

Brain sections were stained by TUNEL staining Kit (Roche Inc., Basel, Switzerland), the TUNEL-positive cells were expressed by fluorescein-dUTP with dNTP or POD with 3-3' diaminobenzidine (DAB) (manufacturer's protocol for in situ Apoptosis Detection Kit [Roche Inc.]) as described previously [13]. A negative control was similarly performed except for omitting TUNEL reaction mixture. Cells showing nuclear condensation/fragmentation and apoptotic bodies in the absence of cytoplasmic TUNEL reactivity, brown staining of nuclei were considered as apoptotic cells. Apoptotic cells were confirmed with the help of a pathologist blinded to the grouping. The number of TUNEL-positive cells in each region was counted in a high-powered field (× 400) by a investigator who was blinded to the studies, and expressed as number/mm^2^. The number of animals used in each group was SAH+ atorvastatin (n = 5), SAH+DMSO (n = 5), SAH (n = 5), and SC (n = 5).

### Cell death assay

For quantification of DNA fragmentation in brain tissue, which indicates apoptotic cell death, a commercial enzyme immunoassay was used to determine cytoplasmic histone-associated DNA fragments (Roche Molecular Biochemicals, USA). Fresh brain tissue was taken and protein extraction of the cytosolic fraction was performed as described [28]. A cytosolic volume containing 50 μg of protein was used for the enzyme linked immunosorbent assay, as per the manufacturer's protocol. The number of animals used in each group was SAH+ atorvastatin group (n = 5), SAH+DMSO group (n = 5), SAH group (n = 5), and SC group (n = 5).

### Western blotting analysis

The method for Western blot has been described previously [29]. The samples (20 μg protein) were separated by sodium dodecyl sulfatepolyacrylamide gel electrophoresis with 10% polyacrylamide gel. The following primary antibodies were used: goat monoclonal anti-caspase-3 (P17, 1: 1000), goat monoclonal anti-caspase-8 (P18), mouse monoclonal anti-AIF (1: 1500), rabbit polyclonal anti-Cytochrome C (1: 1500), rabbit polyclonal anti-Bax, rabbit monoclonal anti-Bcl-2, and rabbit polyclonal anti-β-actin (1: 3000) antibody (Santa Cruz Inc. USA). After incubation with the primary antibodies, the nitrocellulose membranes were washed with TBST dilution and incubated with appropriate horseradish peroxidase-labeled secondary antibodies (1:1000, Santa Cruz Inc. USA) using 1% nonfat milk in TBST for 1 hour at room temperature. After two rinses and four washes with PBS/Nonidet P-40 or TBST, the membranes were incubated in ECL (Amersham, Little Chalfont, U.K.) reagent for HRP (60s) and exposure to autoradiography film for visualization of the bands. The results were quantified by Quantity One Software (Biorad). The number of animals used in each group was SAH+atorvastatin (n = 12), SAH+DMSO (n = 12), SAH (n = 12), and SC (n = 12).

### Quantitative Real-time RT-PCR

Total RNA was extracted from brain samples using TrIzol Regeants (Gibco BRL; Life Technologies, Rockville, MD, U.S.A) as the manufacture's instructions. As a specific internal control, the level of glyceraldehyde phosphate dehydrogenase (GAPDH) was evaluated. To make the standard curve, serially diluted standard plasmids were examined. The reaction mixtures contained diluted cDNA, SYBR Green I Nucleic Acid Stain (Invitrogen Life Technologies, Carlsbad, CA, USA), 20 μM of each gene specific primer and nuclease-free water to a final volume of 50 μl. The PCR reactions were cycled 40 times by a three-step cycle procedure (denaturation 95°C, 15 s; annealing 60°C, 1 min; extension 72°C, 1 min) after initial stages (45°C, 2 min; 95°C, 10 min). All samples were analyzed in triplicate. The primers were as follows:

Caspase-3: Forward: 5'-TGAGGTGCGGAGCTTGGAAC-3'

Reverse: 5'-GAGTCCATCGACTTGCTTCCATG-3'

Caspase-8:Forward:5'-AGGGGAGCTGTGACTGGCG-3'

Reverse:5'-TGGTCCAAGCACAGGAACTTGA-3'

GAPDH:Forward:5'-CGCCCAGAACATCATCCCT-3'

Reverse:5'-GCACTGTTGAAGTCGCAGGAGA-3'

### Statistics

Data were expressed as mean ± S.D. Statistical differences between atorvastatin and other groups were compared by one-way ANOVA and then the Tukey-Kramer multiple comparison procedure, if a significant difference had been determined by ANOVA. Significance of differences in neurologic scores was analyzed by Kruskal-Wallis one-way ANOVA followed by multiple comparison procedures with Dunn's method. Differences in mortality between groups were tested using *Fisher *exact test. Probability value of *P *< 0.05 was considered statistically significant.

## Abbreviations

SAH: subarachnoid hemorrhage; Atorva: Atorvastatin; DMSO: dimethyl sulfoxide; SC: sham control; BBB: blood-brain barrier; AIF: apoptosis inducing factor; H-E: hematoxylin and eosin; PBS: physiological buffer solution.

## Competing interests

The authors declare that they have no competing interests.

## Authors' contributions

GC and LW carried out the Western blotting and Real-time PCR. GC and SD performed the animal model and TUNEL staining. LW and SD carried out the neurological deficits. SG and LZ designed the experiment oversaw all components including manuscript preparation. All authors read and approved the final manuscript.
